# Unraveling the clinicopathological features driving the emergence of *ESR1* mutations in metastatic breast cancer

**DOI:** 10.1038/s41523-018-0075-5

**Published:** 2018-08-02

**Authors:** Yanan Kuang, Bilal Siddiqui, Jiani Hu, Matthew Pun, MacIntosh Cornwell, Gilles Buchwalter, Melissa E. Hughes, Nikhil Wagle, Paul Kirschmeier, Pasi A. Jänne, Cloud P. Paweletz, Nancy U. Lin, Ian E. Krop, William T. Barry, Eric P. Winer, Myles Brown, Rinath Jeselsohn

**Affiliations:** 1 Belfer Center for Applied Cancer Science, Dana-Farber Cancer Institute, Boston, MA 02215 USA; 2 Department of Medical Oncology, Dana Farber-Cancer Institute, Boston, MA 02215 USA; 3 Beth Israel Deaconess Medical Center, Boston, MA 02215 USA; 4 Department of Biostatistics & Comp Biology, Dana-Farber Cancer Institute, Boston, MA 02215 USA; 5 Center for Functional Cancer Epigenetics, Dana Farber-Cancer Institute, Boston, MA 02215 USA; 6 Breast Oncology Center, Dana-Farber Cancer Institute, Boston, MA 02215 USA; 7 Lowe Center for Thoracic Oncology, Dana-Farber Cancer Institute, Boston, MA 02215 USA

## Abstract

*ESR1* mutations were recently found to be an important mechanism of endocrine resistance in ER-positive (ER + ) metastatic breast cancer. To determine the clinicopathological features driving the emergence of the *ESR1* mutations we studied plasma cfDNA and detailed clinical data collected from patients with metastatic breast cancer. Droplet Digital PCR was performed for the detection of the most common *ESR1* mutations and *PIK3CA* mutations. Among the patients with ER + /HER2- disease, *ESR1* mutations were detected in 30% of the patients. There were no associations between the pathological features of the primary disease or time to distant recurrence and the emergence of *ESR1* mutations in metastatic disease. The prevalence of the *ESR1* mutations was significantly associated with prior treatment with an aromatase inhibitor in the adjuvant or metastatic setting. The prevalence of the *ESR1* mutations was also positively associated with prior fulvestrant treatment. Conversely, the prevalence of *ESR1* mutations was lower after treatment with a CDK4/6 inhibitor. There were no significant associations between specific systemic treatments and the prevalence of *PIK3CA* mutations. These results support the evolution of the *ESR1* mutations under the selective pressure of treatment with aromatase inhibitors in the adjuvant and metastatic settings and have important implications in the optimization of adjuvant and metastatic treatment in ER + breast cancer.

## Genetics: Drug treatment spurs new resistance mutations

Treatment with aromatase inhibitors, a class of drugs that suppress the synthesis of estrogen, can drive the evolution of mutations in the estrogen receptor gene *ESR1*, leading to tumor resistance against hormone therapies. To better understand the emergence of *ESR1* mutations, Rinath Jeselsohn from the Dana-Farber Cancer Institute  in Boston, Massachusetts, USA, and coworkers tested tumor DNA contained within blood samples from 155 women with metastatic breast cancer. They found *ESR1* mutations rarely in women with any molecular subtype of cancer other than estrogen receptor-positive disease. Nothing about the primary tumor predicted who would develop *ESR1* mutations; however, treatment with an aromatase inhibitor was associated with mutations arising. The findings highlight the need to develop therapeutic regimens that reduce the selective pressure for *ESR1* mutations and/or target these mutations directly.

## Introduction

The *ESR1* ligand-binding domain (LBD) mutations were unveiled in recent years as an important mechanism of acquired endocrine resistance that evolves under the selective pressure of endocrine treatments. These mutations are rarely found in primary estrogen receptor-positive (ER + ) breast cancers but have a high prevalence in metastatic disease and lead to constitutive ligand independent activity.^1–3^ The most prevalent mutations as detected in a number of studies are the Y537S and D538G mutations. The third most common mutation is the E380Q mutation, also located in the LBD.^4^

Liquid biopsies detecting circulating tumor DNA (cfDNA) are emerging as a useful non-invasive tool for serial monitoring of genomic alterations in patients with metastatic cancer. Multiple studies have now shown that the *ESR1* LBD mutations can be successfully detected in the plasma of patients with metastatic ER + breast cancer.^5,6^ Patients with ER + metastatic breast cancer who received an aromatase inhibitor (AI) in the metastatic setting compared to AI naive patients had a higher prevalence of cfDNA *ESR1* mutations.^7^ Moreover, patients with metastatic ER + breast cancer with detectable cfDNA *ESR1* mutations had decreased progression free survival on subsequent treatment with an aromatase AI.^6^

In this study, we sought to comprehensively study the associations between the emergence of the *ESR1* mutations in cfDNA, clinicopathological features, and treatments in the adjuvant and metastatic settings. We prospectively collected plasma samples from patients with metastatic breast cancer from a single institution and tested for the most common *ESR1* mutations using droplet digital PCR (ddPCR). We also tested for the most common *PIK3CA* mutations, as *PIK3CA* mutations have been reported to be an early event in ER + breast cancer and are found in more than 30% of ER + primary treatment naive breast cancers. The frequency of *PIK3CA* mutations do not change under the pressure of endocrine treatments or the development of endocrine resistance and metastatic disease.^2,8^

## Results

### Patient and sample characteristics

We prospectively collected 155 plasma samples from patients with metastatic breast cancer enrolled on this biospecimen collection protocol. Median age at initial breast cancer diagnosis was 46 years, (range 29–81 years). Subtype distribution was as follows: ER + /HER2-, *n* = 113 (73%); HER2 + (either ER + or ER-), *n* = 25 (16%); ER and PR-negative/HER2-negative (triple-negative breast cancer, TNBC), *n* = 17 (11%). Results of clinical ER, PR, and HER2 testing on an archival metastatic biopsy specimen were available in 147 (95%) of the 155 patients included in this study.

Patient and sample characteristics of the ER + /HER2- cohort are shown in Table 1. Among patients who presented with stage I–III disease, median disease-free interval was 58.9 months (range 0–368.8). Among the 113 patients presenting with hormone receptor-positive/HER2-negative breast cancer, the primary tumor was ER and PR-positive in 92 (81.4%) of patients; 15 (13.3%) patients had ER-positive/PR-negative disease. Approximately one-fifth of the patients (*n* = 21) presented with de novo metastatic disease. Among patients who presented with stage I–III ER and/or PR-positive disease, 84% received adjuvant endocrine therapy, and median disease-free interval was 67.4 months (range 6.1–312.1). Contemporaneous research metastatic biopsies were available for 23 of the 113 patients.

**Table 1:** Characteristics of the ER + /HER2-cohort at time of diagnosis of primary disease

Characteristic	All patients (113)	*ESR1* mutant (34)	*ESR1* WT (79)	*p*-values	*PIK3CA* mutant (36)	*PIK3CA* WT (77)	*p*-values
Median age at random assignment, years (IQR)	57.1 (48.7, 65.1)	57.1 (52.8, 68.1)	56.7 (46.7, 64.6)	0.16	57.9 (49.1, 63.4)	56.7 (48.4, 65.1)	0.61
Tumor grade							
I	12 (10.6%)	6 (17.6%)	6 (7.6%)	0.19	6 (16.7%)	6 (7.8%)	0.19
II	50 (44.2%)	15 (44.1%)	35 (44.3%)		15 (41.7%)	35 (45.4%)	
III	43(38.1%)	10 (29.4%)	33 (41.8%)		13 (36.1%)	30 (39.0%)	
Unknown	8 (7.1%)	3 (8.8%)	5 (6.3%)		2 (5.5%)	6 (7.8%)	
Stage							
0	1 (0.9%)	0 (0%)	1 (1.3%)	0.022	0 (0%)	1 (1.3%)	0.48
I	23 (20.4%)	13 (38.2%)	10 (12.7%)		10 (27.8%)	13 (16.9%)	
II	52 (46.0%)	14 (41.2%)	38 (48.1%)		15 (41.7%)	37 (48.0%)	
III	10 (8.8%)	4 (11.8%)	6 (7.6%)		5 (13.9%)	5 (6.5%)	
IV	21 (18.6%)	3 (8.8%)	18 (22.8%)		6 (16.7%)	15 (19.5%)	
Unknown	6 (5.3%)	0 (0%)	6 (7.6%)		0 (0%)	6 (7.8%)	
PR status-primary							
Positive	92 (81.4%)	27 (79.4%)	65 (82.3%)	0.77	30 (83.3%)	62 (80.5%)	0.38
Negative	15 (13.3%)	5 (14.7%)	10 (12.7%)		3 (8.3%)	12 (15.6%)	
Unkown	6 (5.3%)	2 (5.9%)	4 (5.1%)		3 (8.3%)	3 (3.9%)	

### *ESR1* and *PIK3CA* mutations detected in cfDNA of patients with metastatic breast cancer are highly concordant with metastatic tumor samples

**Fig. 1:**
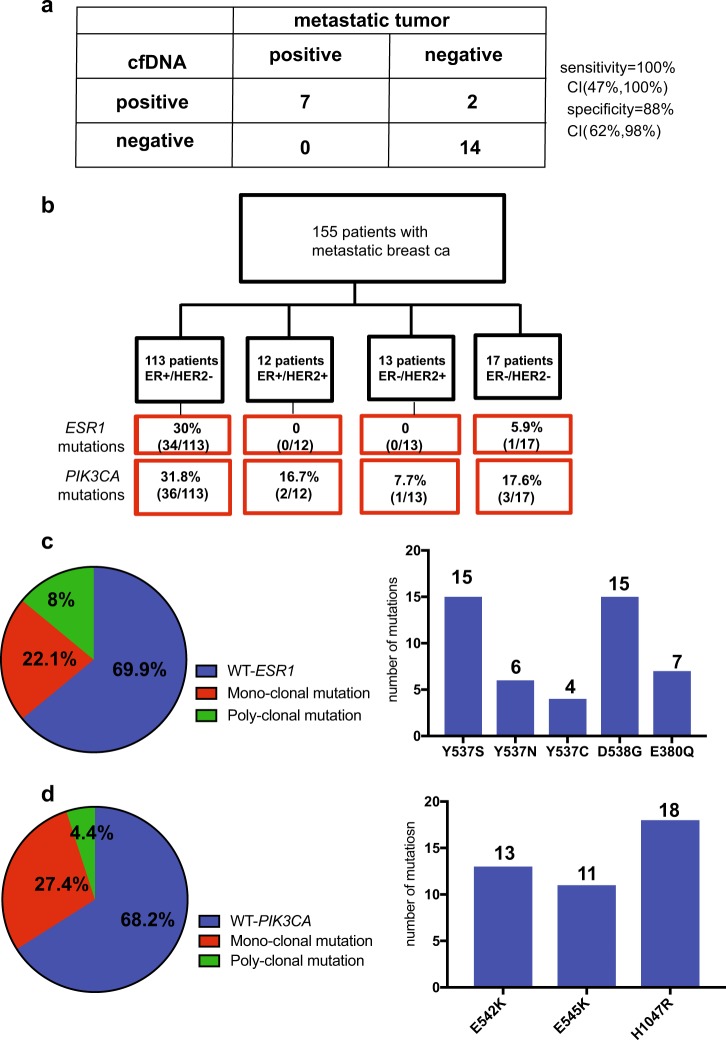
Prevalence of cfDNA *ESR1* and *PIK3CA* mutations in patients with metastatic breast cancer. **a** The sensitivity and specificity of mutation detection in cfDNA compared to paired metastatic tissue samples. **b** REMARK diagram. **c** Prevalence of *ESR1* mutations in cfDNA among ER + /HER2-metastatic patients. **d** Prevalence of *PIK3CA* mutations in cfDNA among ER + /HER2- metastatic patients

We developed a highly sensitive assay using droplet digital PCR (ddPCR) for the detection of the most common *ESR1* (Y537S, D538G, E380Q, Y537N, Y537C) and *PIK3CA* mutations (H1047R, E542K, E545K). To examine the sensitivity and specificity of *ESR1* mutant detection in cfDNA compared to detection in tissue biopsies both tested by ddPCR, we tested for the *ESR1* mutations in a subset of 23 patients from whom contemporaneous metastatic tumor biopsies were available. Seven *ESR1* mutations were found in the tissue samples and all were detected by the cfDNA analysis. There were two mutations detected by the cfDNA analysis that were not detected in the tumor samples, which likely reflects the ability of the cfDNA test to capture information from genetically heterogeneous metastatic samples. Overall, the plasma and metastatic tumor samples were highly concordant with 100% sensitivity and 88% specificity for the plasma *ESR1* cfDNA assay compared to testing metastatic tissue samples applying ddPCR (Fig. 1a). High concordance between cfDNA ESR1 mutations and metastatic tissues, was seen in previous studies.^6,9^

### *ESR1* and *PIK3CA* mutations are frequently detected in cfDNA of patients with metastatic breast cancer

Given the concordance between ddPCR in blood vs. tumor specimens, we next proceeded to evaluate the frequencies of *ESR1* mutations and *PIK3CA* mutations in the entire cohort (*n* = 155). We found 35 (22.6%) and 42 (27%) patients to have at least 1 *ESR1* mutation and 1 *PIK3CA* mutation, respectively. All patients with an *ESR1* mutation had ER + disease, with the exception of one patient with TNBC (Fig. 1b). This patient was diagnosed with TNBC based on pathology of the primary and a metastatic lesion. The cfDNA ESR1 mutation was confirmed in replicate assays. This patient did not have a history of ER + breast cancer and this mutation likely reflects a sub-clone of ER + cells that is a rare event as in previous studies the *ESR1* recurrent mutations were not detected in TNBC metastatic tissue samples.^2^ In the subgroup of patients with ER + /HER2-metastatic breast cancer, 30% were found to have an *ESR1* mutation and 26% of these patients had more than one *ESR1* mutation suggesting polyclonal disease (Fig. 1c). Similar to previous reports, the most common mutations were the Y537S (15 or 31.9%) and D538G (15 or 31.9%) mutations followed by the E380Q mutation (7 or 14.9%). In contrast to the *ESR1* mutations, the *PIK3CA* mutations were not significantly more prevalent in ER + /HER2-disease compared to other breast cancer subtypes and 14% of the *PIK3CA* mutant samples had more than one *PIK3CA* mutation (Fig. 1d). The presence of *ESR1* and *PIK3CA* mutations were not independent of each other. Among the 113 patients with ER + disease, patients without a detectable *PIK3CA* mutation were less likely to have an *ESR1* mutation (21% of the patients with WT *PIK3CA* had a *ESR1* mutation, whereas 50% of the patients with a *PIK3CA* mutation also had an *ESR1* mutation, *p*-value = 0.002).

### The E380Q mutation confers ligand independent cell growth and relative resistance to tamoxifen and fulvestrant

The three most commonly reported ER LBD mutations are the D538G, Y537S, and the E380Q mutations; our data confirm these findings. The D538G and Y537S mutations are both within the C-terminal helix within helix 12 and their structure has been solved by crystallography studies.^10,11^ In addition, a number of studies have investigated the functional roles of these mutations.^12^ The structure of the E380Q mutation has not been solved. However, since the E380 residue is in the vicinity of helix 12, it is highly plausible that this mutation has similar functional characteristics. To date, only a limited number of studies have investigated the functional consequences of the E380Q mutation and the data are conflicting. In one study, a PDX model harboring this mutation did not display E2 independent tumor growth, whereas overexpression of the E380Q mutation in MCF7 cells resulted in E2 independent transcriptional activity and proliferation, albeit to a lesser extent compared to cells overexpressing the Y537S mutation.^4,13^

**Fig. 2:**
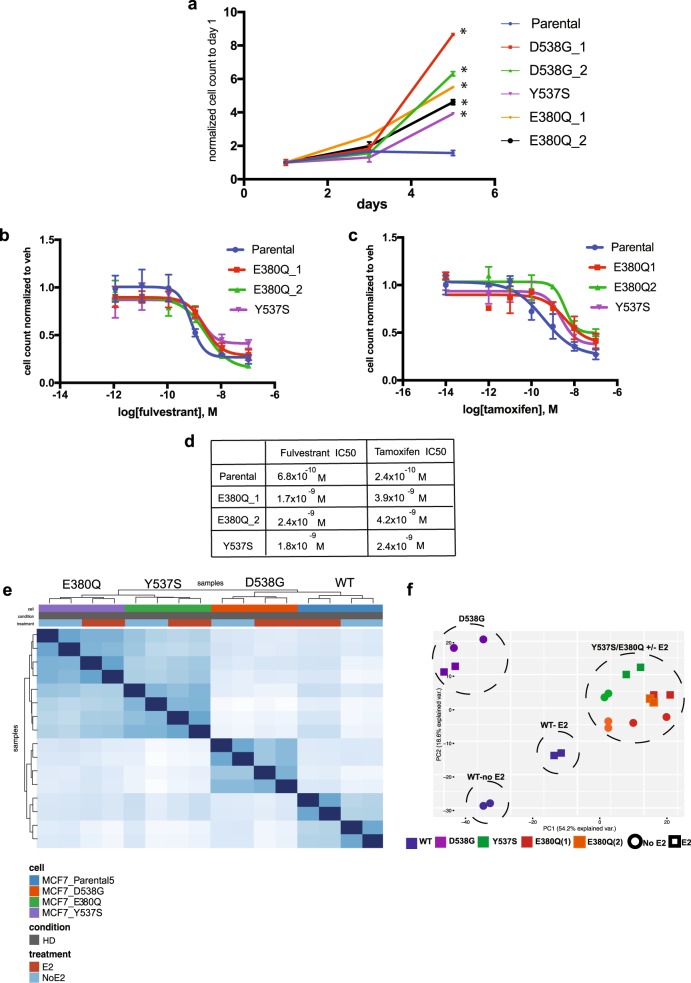
The E380Q mutation confers ligand independent cell growth and relative resistance to tamoxifen and fulvestrant. **a** Cell growth of the E380Q (clone 1 and 2), Y537S and D538G (clone 1 and 2) knocked-in MCF7 cells and parental MCF7 cells in hormone-depleted (HD) conditions. Error bars represent S.D., *n* = 3. *p-value < 0.001. **b** Dose–response curves of the MCF7 parental cells, E380Q (clones 1 and 2) and Y537S cells with fulvestrant treatment on day 5. Error bars represent S.D., *n* = 3. **c** Dose–response curves of the MCF7 parental cells and E380Q cells (clones 1 and 2) with tamoxifen treatment on day 5. Error bars represent S.D., *n* = 3. **d** IC50 values for fulvestrant and tamoxifen in MCF7 parental cells and E380Q knocked-in MCF7 cells (clones 1 and 2). **e** Pairwise correlation analysis of the RNA-seq in HD and HD + Estradiol (E2) conditions. **f** Principal component analysis of the transcriptomes of *ESR1* WT, D538G, Y537S, and E380Q mutant cells

To study the functional roles of the E380Q mutation, we generated and characterized single allele E380Q, Y537S, and D538G knock-in MCF7 cells. Similar to the Y537S and D538G knocked-in MCF7 cells, the E380Q knocked-in cells displayed a significant growth advantage in hormone-depleted (HD) conditions when compared to the MCF7 WT *ESR1* parental cells (Fig. 2a). In addition, the E380Q knocked-in cells were relatively resistant to tamoxifen and fulvestrant similar to the Y537S mutant cells (Fig. 2b–d). To study the global transcriptional effects of the E380Q mutation, we performed RNA-seq in the knock-in cell lines. Pairwise correlation analysis of the RNA-seq in HD and HD + Estradiol (E2) clustered the E380Q and Y537S mutant cells together and distinctly separated from the D538G mutant and the WT-ER cells (Fig. 2e). Similarly, in a principal components analysis (Fig. 2f), the principal component 1 (PC1) is driven by the *ESR1* allele with the E380Q allele clustering with Y537S and the WT and D538G alleles were in distinct clusters. The medium conditions resolved along PC2 and distinctly segregated the WT cells in HD conditions from HD + E2 conditions. This distinct separation between the HD and HD + E2 conditions was not seen in the mutant knock-in cells. These results indicate that the E380Q mutation, similar to the Y537S and D538G mutation, exhibits constitutive transcriptional activity, ligand independent cell proliferation, and relative resistance to tamoxifen and fulvestrant. Previous studies have demonstrated that the Y537S and D538G mutations confer distinct transcriptional programs.^14,15^ Here, we show that the E380Q mutant transcriptional program resembles the transcriptional program of the Y537S mutant and both are different from the D538G mutant. Taken together, these results support the inclusion of the E380Q *ESR1* allele as an important resistance mutation.

### *ESR1* and not *PIK3CA* mutations are associated with presenting clinicopathological features and sites of metastasis in ER + /HER2-metastatic disease

To examine the pathological and clinical features associated with cfDNA *ESR1* and *PIK3CA* mutations in the 113 ER + /HER-cases, we looked at differences in the clinical features of the breast cancer between patients who had and did not have detectable *ESR1* or *PIK3CA* mutations in cfDNA. There were no significant associations between the pathological features of the primary tumor and cfDNA detection of *ESR1* or *PIK3CA* mutations. Features examined included tumor grade, stage (I, II, or III) and PR status (Table 1). In contrast, there was a significant positive association between PR expression in the metastasis and *ESR1* mutations (*p*-value = 0.006), but not *PIK3CA* mutations (*p*-value = 0.22), potentially indicative of active ER transcription in the presence of the *ESR1* mutations (Table 2).

**Table 2:** Patient characteristics of ER + /HER2-cohort after diagnosis of metastatic disease

Characteristic	All patients (113)	*ESR1* mutant (34)	*ESR1* WT (79)	*p*-values	*PIK3CA* mutant (36)	*PIK3CA* WT (77)	*p*-values
PR metastatic							
Positive	47 (41.6%)	22 (64.7%)	25 (31.6%)	0.006	19 (52.8%)	28 (36.4%)	0.22
Negative	59 (52.2%)	12 (35.3%)	47 (59.5%)		17 (47.2%)	42 (54.5%)	
Unknown	7 (6.2%)	0 (0%)	7 (8.9%)		0 (0%)	7 (9.1%)	
Presentation of metastatic disease							
Relapsed	86 (76.1%)	31 (91.2%)	55 (69.6%)	0.016	29 (80.6%)	57 (74.0%)	0.5
De novo	27 (23.9%)	3 (8.8%)	24 (30.4%)		7 (19.4%)	20 (26.0%)	
Site of mets							
Liver							
Yes	74 (65.5%)	30 (88.2%)	44 (55.7%)	0.001	26 (72.2%)	48 (62.3%)	0.4
No	38 (33.6%)	4 (11.8%)	34 (43.0%)		10 (27.8%)	28 (36.4%)	
Bone							
Yes	87 (77.0%)	33 (97.1%)	54 (68.4%)	< 0.001	32 (88.9%)	55 (71.4%)	0.056
No	25 (22.1%)	1 (2.9%)	24 (30.4%)		4 (11.1%)	21 (27.3%)	
Lung							
Yes	38 (33.6%)	13 (38.2%)	24 (30.4%)	0.51	12 (33.3%)	25 (32.5%)	1.00
No	75 (66.4%)	21 (61.8%)	54 (68.4%)		24 (66.7%)	51 (66.2%)	
Visceral disease							
Yes	69 (61.1%)	25 (73.5%)	44 (55.7%)	0.2	26 (72.2%)	43 (55.8%)	0.21
No	40 (35.4%)	9 (26.5%)	31 (39.2%)		10 (27.8%)	30 (39.0%)	
Unknown	4 (3.5%)	0 (0%)	4 (5.1%)		0 (0%)	4 (5.2%)	
Status of metastatic disease							
New diagnosis of mets	35 (31.0%)	3 (8.8%)	32 (40.5%)	< 0.001	9 (25.0%)	26 (33.8%)	0.002
Stable disease	14 (12.4%)	1 (2.9%)	13 (16.5%)		0 (0%)	14(18.2%)	
Progressive disease	63 (55.8%)	30 (88.2%)	33 (41.8%)		27 (75.0%)	36 (46.8%)	
Unknown	1 (0.9%)	0 (0%)	1 (1.3%)		0	1	

**Fig. 3:**
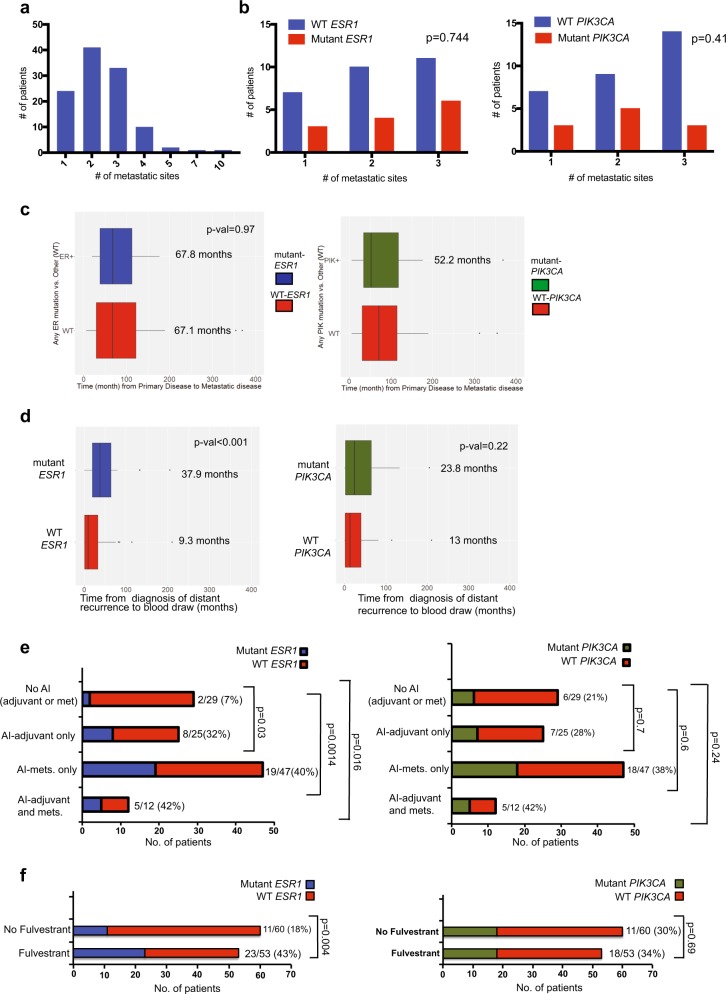
Associations between clinical features and *ESR1* mutations. **a** The distribution of the patients based on number of metastatic sites for all 113 ER + /HER2-patients. **b** Associations between the number of metastatic sites and the prevalence of *ESR1* or *PIK3CA* mutations. **c** Interval time from diagnosis of primary disease and distant recurrence in patients with and without *ESR1* mutations and in patients with and without *PIK3CA* mutations. Center line is median, box limits are the upper and lower quartiles, whiskers are the 1.5 x inter-quartiles and the points are the outliers. **d** Interval time from diagnosis of distant recurrence to blood draw in patients with and without *ESR1* mutations and in patients with and without *PIK3CA* mutations. Center line is median, box limits are the upper and lower quartiles, whiskers are the 1.5 x inter-quartiles and the points are the outliers. **e** Associations between aromatase inhibitor (AI) treatment and the prevalence of *ESR1* and *PIK3CA* mutations. **f** Associations between fulvestrant treatment and the prevalence of *ESR1* and *PIK3CA* mutations

While there was no association between visceral vs. bone disease only or the number of metastatic sites and the presence of cfDNA *ESR1* or *PIK3CA* mutations, patients with liver and bone metastases were more likely to have detectable cfDNA *ESR1* but not *PIK3CA* mutations (*p*-value = 0.001 for liver and *p*-value < 0.001 for bone metastases) (Fig. 3a-3b and Table 2). We have detected an association between the D538G mutations and liver metastases in previous studies.^15^ In this study, the association between the *ESR1* mutations and liver metastases is seen when including all the allelic LBD mutations tested here and not specifically the D538G mutation. This is possibly due to the fact that cfDNA is capturing mutations from multiple metastatic sites and not specifically the liver. The mechanism of this organotropism remains to be elucidated.

### *ESR1* mutation frequency varies according to disease status, timing of blood draw and emerge after aromatase inhibitor treatment in the adjuvant and metastatic settings

In this study, a convenience blood sample was collected after study enrollment at any point in a patient’s metastatic disease course. We thus evaluated the relationship between the timing of blood collection relative to disease status and endocrine therapy, and the frequency of *ESR1* and *PIK3CA* alterations. The clinical status of the metastatic disease at the time of the plasma collection was strongly linked to the detection of *ESR1* and *PIK3CA* mutations in cfDNA (Table 2). Patients who had stable disease or a new diagnosis of metastatic disease at the time of the blood draw were less likely to have cfDNA *ESR1* (11.7%) or *PIK3CA* (25%) mutations compared to patients with progressive disease (88.2% for *ESR1* mutations and 75% for *PIK3CA* mutations, respectively) at the time of the blood draw. This finding was seen for mutations in both genes and is likely reflective of less shedding of cfDNA at the time of stable disease or new diagnosis of metastases, as was shown for other mutations and cancer types.^16–18^

The disease-free interval defined as the time from the diagnosis of primary disease to the development of distant recurrence did not significantly differ in patients who had or did not have detectable cfDNA *ESR1* or *PIK3CA* mutations (Fig. 3c). Conversely, cfDNA *ESR1* mutation frequency positively correlated with the duration of time elapsed from the diagnosis of distant recurrence to the plasma collection (average time 9.3 months for WT *ESR1* and 37.9 months for mutant *ESR1*, *p*-value < 0.001) (Fig. 3d). In addition, patients who had relapsed metastatic disease were more likely to have detectable cfDNA *ESR1* mutations (*p*-value = 0.016 for *ESR1)*, compared to patients who presented with de novo metastatic breast cancer (Table 2). These two associations were not seen with cfDNA *PIK3CA* mutations and may be the consequence of more prolonged selection by endocrine therapies.

To test this hypothesis, we next investigated the impact of systemic treatments given prior to the blood sample collection on the prevalence of *ESR1* and *PIK3CA* mutations in cfDNA in the ER + /HER2-cohort. Chemotherapy treatment in the setting of neoadjuvant/adjuvant treatment for early-stage disease or metastatic disease was not associated with the prevalence of *ESR1* or *PIK3CA* mutations in cfDNA (Table 3). The majority of the patients were exposed to treatment with an AI (*n* = 84) either in the adjuvant, metastatic setting or both prior to the blood draw. Patients who received an AI at any time compared to patients who did not receive AI treatment were more likely to have detectable *ESR1* mutations in cfDNA (Fig. 3e). We next examined whether the timing of AI treatment influenced the prevalence of the *ESR1* and *PIK3CA* mutations. We observed high rates of *ESR1* mutations among patients with metastatic breast cancer, irrespective of whether AI exposure was in the adjuvant setting only (32%, *p*-value = 0.03), metastatic setting only (40%, *p*-value = 0.0014), or in both the adjuvant and metastatic settings (42%, *p*-value = 0.016). There was no significant difference in the prevalence of *ESR1* mutations in patients who received AI treatment in the adjuvant setting only vs. patients who received AI treatment for metastatic disease only (*p*-value = 0.62). Notably, given the proposed role of the PI3K pathway in mediating endocrine resistance, there was no association between AI exposure in the adjuvant, metastatic or both and *PIK3CA* mutations (*p*-value > 0.05 for all treatment settings), supporting a selection effect of AI treatment unique to *ESR1* mutations. We next studied the association of fulvestrant treatment for metastatic disease and *ESR1* and *PIK3CA* mutations. Similar to AI treatment, we observed an increase in the prevalence of the *ESR1* mutations in patients who received fulvestrant compared to those who did not (*p*-value = 0.004). Again, this association was not seen for the *PIK3CA* mutations (*p* = 0.69) (Fig. 3f). We also tested the associations between tamoxifen treatment and the *ESR1* mutations. Overall 49 patients received either single agent adjuvant tamoxifen or sequential treatment with tamoxifen followed by an aromatase inhibitor. To look at the effect of adjuvant tamoxifen treatment on the emergence of the *ESR1* mutations, we selected patients that received adjuvant tamoxifen and no endocrine treatment (AI or fulvestarnt) in the metastatic setting prior to the blood draw. Among these patients, six patients received sequential adjuvant tamoxifen followed by an AI and one patient out of these patients was found to have an *ESR1* mutation. There were nine patients that received adjuvant tamoxifen only and of these patients, one patient was found to have an *ESR1* mutation. There was no significant positive or negative associations between adjuvant tamoxifen treatment (adjuvant tamoxifen/adjuvant tamoxifen followed by an AI) and the emergence of the *ESR1* mutations (Supplementary Fig. S1). This analysis was limited because of the small numbers of patients and should be interpreted with caution. Nonetheless, these results demonstrate that the *ESR1* mutations are found in a small number of patients that received prior endocrine treatment with tamoxifen alone, albeit in small numbers. In the metastatic setting, 20 patients received tamoxifen treatment prior to the blood draw. We did not detect a significant association between the tamoxifen treatment for metastatic disease and the prevalence of the *ESR1* mutations. Collectively, these results support the clonal evolution unique to the *ESR1* mutations under the selective pressure of AI treatment in the adjuvant and metastatic settings, and fulvestrant treatment.

**Table 3:** Associations between treatments and mutations in ER + /HER2-cohort

Treatment	All patients (113)	*ESR1* mutant (34)	*ESR1* WT (79)	*p*-values	*PIK3CA* mutant (36)	*PIK3CA* WT (77)	*p*-values
Neo/adj chemotherapy							
Yes	68 (60.2%)	24 (70.6%)	44 (55.7%)	0.15	20 (55.6%)	48 (62.3%)	0.54
No	45 (39.8%)	10 (29.4%)	35 (44.3%)		16 (44.4%)	29 (37.7%)	
Chemotherapy in metastatic disease							
Yes	61 (54.0%)	23 (67.6%)	38 (48.1%)	0.067	19 (52.8%)	42 (54.5%)	1.00
No	52 (46.0%)	11 (32.4%)	41 (51.9%)		17 (47.2%)	35 (45.5%)	
Palbociclib							
Yes	23 (20.4%)	2 (5.9%)	21 (26.6%)	0.011	5 (13.9%)	18 (23.4%)	0.32
No	90 (79.6%)	32 (94.1%)	58 (73.4%)		31 (86.1%)	59 (76.6%)	
Everolimus							
Yes	16 (14.2%)	7 (20.6%)	9 (11.4%)	0.24	4 (11.1%)	12 (15.6%)	0.77
No	97 (85.8%)	27 (79.4%)	70 (88.6%)		32 (88.9%)	65 (84.4%)	

### *ESR1* alterations in the setting of novel targeted agents

A subset of patients in this study received a CDK4/6 inhibitor (*n* = 23) or everolimus (*n* = 16) prior to collection of the blood specimen for cfDNA. Interestingly, patients who received palbociclib for metastatic disease were less likely to have detectable *ESR1* mutations in cfDNA but this was not the case for *PIK3CA* mutations (*p*-value = 0.01) (Table 3). In contrast, there was no association between everolimus treatment in metastatic disease and the prevalence of *ESR1* or *PIK3CA* mutations in cfDNA (Table 3). These results suggest that palbociclib treatment may be effective in inhibiting tumors harboring the *ESR1* mutations, which is in line with previous reports demonstrating that the tumors harboring the *ESR1* mutation are sensitive to palbociclib.^19^

## Discussion

Previous studies have shown that the *ESR1* endocrine resistance mutations are rarely detected in primary treatment naive tumors and evolve predominantly in metastatic disease under the selective pressure of endocrine treatment. cfDNA analysis using ddPCR is emerging as a non-invasive highly sensitive test that can capture the heterogeneity of the mutational landscape from multiple metastatic sites. In this study, we used cfDNA to comprehensively study the clinicopathological features and treatments associated with the evolution of the *ESR1* mutations. Since these mutations drive endocrine therapy resistance and confer worse outcomes in metastatic disease,^20^ understanding these associations is clinically important.

In line with previous studies, we observed a strong link between treatment with aromatase inhibitors and the evolution of the *ESR1* mutations. Importantly, in our study the prevalence of the *ESR1* mutations increased equally after AI treatment in the adjuvant setting, metastatic setting or both. The finding that the *ESR1* mutations emerge during adjuvant AI treatment differs from an earlier study and has important clinical implications.^6^ This finding may explain why adjuvant AI in comparison to adjuvant tamoxifen reduces the risk of distant recurrence, but in most studies has not been shown to significantly improve overall survival.^21^ This finding also highlights the importance of improving adjuvant endocrine treatments to reduce the clonal selection of the *ESR1* mutations, particularly for patients at high risk of recurrence.

In a previous study, the emergence of the *ESR1* mutations was seen with AI treatment in the metastatic setting only.^6^ The reason for this discrepancy between our study and the study by Schiavon et al. is not clear. This may be due to the overall small number of patients in both studies. Another possible explanation for the discrepancy is differences between the two study centers in the rates of adherence to adjuvant aromatase inhibitors. Non-adherence to adjuvant aromatase inhibitors is substantial and in the range of 30%,^22,23^ which could account for the discordant results. Nonetheless, a larger study to resolve this inconsistency is needed.

Notably, we did not find significant correlations between the detection of cfDNA *ESR1* mutations in metastatic disease and clinical or pathological features at the time of initial presentation of early-stage ER + disease. In addition, chemotherapy treatment, either adjuvant or for metastatic diseases, did not influence the prevalence of the *ESR1* mutations. The emergence of the *ESR1* mutations was also not linked to the timing of distant recurrence (i.e., early vs. late recurrences). Conversely, the likelihood of detecting *ESR1* cfDNA mutation was increased the longer the duration of ER + metastatic disease. This finding underscores the concept that *ESR1* mutations occur under the selective pressure of endocrine therapy, and highlight the need for improved treatments targeting these ER mutations both to circumvent tumor resistance and effectively treat it when it occurs.

The *PIK3CA* mutations are relatively common in ER + breast cancers and are an early mutational event in the development of breast cancer.^24^ Previous reports have indicated that the prevalence of the *PIK3CA* mutations in primary and metastatic tumors is comparable and these mutations likely do not evolve under the selective pressure of current treatments.^2^ In this study, we analyzed the most common *PIK3CA* mutations in addition to the *ESR1* mutations. Despite the putative role of the PI3K pathway in mediating endocrine resistance, we did not observe any enrichment of *PIK3CA* mutations in patients according to prior endocrine therapy exposure. The strength of our results compared to existing data using metastatic tumor specimens (which are often collected at or near the time of metastatic recurrence) is the span of timing of blood sample collection, with 55% of samples collected ≥ 12 months from the date of metastatic recurrence, suggesting that even with long-term endocrine selection, *PIK3CA* mutations are not selected.

Finally, we report on an intriguing association between the use of CDK4/6 inhibitors and reduced emergence of *ESR1* mutations, though our observations are based on small numbers of patients. Of note, we recently showed in preclinical studies that breast cancers harboring the *ESR1* mutations remain sensitive to palbociclib.^15^ Overall survival data from multiple large, randomized, phase 3 studies evaluating the role of CDK4/6 inhibitors in ER-positive metastatic breast cancer are not yet mature. However, if an OS advantage is eventually demonstrated, our data would suggest a potential mechanism for this, i.e., suppression of emerging *ESR1* mutations that may be associated with resistance to future lines of endocrine therapy, and which would support their preferential use in the first-line setting. In addition, our data would support ongoing trials evaluating the role of CDK4/6 inhibitors in the adjuvant setting (NCT01864746, NCT02513394, and NCT03078751).

Our study has a number of limitations. Because of the relatively small number of patients, we analyzed the different *ESR1* mutations together. However, a number of studies showed that the *ESR1* mutations have allele-specific functional characteristics^14,15^ In addition, the preclinical studies characterizing the E380Q mutation included cell clones derived from one cell line. Lastly, because of the relatively small number of patients we could not perform a meaningful multivariate analysis and, therefore, we could not test for confounding factors. Despite these limitations, our study illuminates the significance of the E380Q mutation, the high prevalence of the *ESR1* mutations in metastatic ER + breast cancer, the fidelity of cfDNA testing for the *ESR1* mutations and the unique evolution of the *ESR1* mutations under the selective pressure of AI treatment in the adjuvant and metastatic settings.

## Methods

### Patients and samples

Patients presenting to the breast oncology clinic at Dana-Farber Cancer Institute were approached for participation in an IRB-approved, prospective protocol, which included clinical data collection, a research biopsy, serial blood collection, and permission for genomic analysis. The Dana-Farber/Harvard Cancer Center (DF/HCC) institutional review board (IRB) approved the protocol (DF/HCC IRB #05-246). The protocol was later amended to include blood collection for ddPCR; hence, though nearly all patients enrolled in the protocol underwent a biopsy as part of the study, only in a subset of patients was the research biopsy contemporaneous with the blood sample collected for ddPCR. Patients provided written informed consent prior to the initiation of any study procedures.

Blood samples for ddPCR were collected from 10/2014 through 9/2016. Pathology of the primary tumors for all patients was reviewed at the Dana Farber Cancer Institute as part of routine clinical care. ER, progesterone receptor (PR), and human epidermal growth receptor 2 (HER2) status were assessed based on the ASCO/CAP guidelines.^25,26^ Breast cancer subtypes (ER + /HER2-, ER + /HER2 + , ER-/HER2 + and triple-negative) were assigned based on the receptor status of the primary tumors.

### Circulating-free DNA extraction

Whole venous blood (6–10 mL) was collected from patients into EDTA lavender capped vacutainer tubes (*Becton and Dickinson)*. Blood was processed within 2 h of collection. Whole blood was centrifuged for 10 min at 1200 × *g* and the plasma supernatant was further cleared by centrifugation for 10 min at 3000 × *g*. Cleared plasma was stored in cryostat tubes at –80 °C until use. Cell-free DNA was isolated using the QIAmp Circulating Nucleic Acid Kit (Qiagen) according to the manufacturer’s protocol. DNA was eluted in AVE buffer (100 μL) and stored at –80 °C until use.

### Droplet digital PCR materials

Droplet digital PCR reagents were ordered from Bio-Rad. Primer/probe mix for *ESR1* and *PIK3CA* mutations were custom-made by Life Technologies. The allele-specific MGB probes are labeled with either VIC or FAM at the 5' end and a nonfluorescent quencher (NFQ) at the 3' end.

The sequences used are:

*ESR1* E380Q: forward primer, 5'-TGGATTTGACCCTCCATGATCAG-3',

reverse primer, 5'-AGACGAGACCAATCATCAGGATCT-3';

probe sequences: 5'-VIC-CCACCTTCTAGAATGTG-MGB-NFQ-3',

5'-FAM-CCACCTTCTACAATGTG-MGB-NFQ-3'.

*ESR1* Y537C: forward primer, 5'-CAGCATGAAGTGCAAGAACGT-3',

reverse primer, 5'-TGGGCGTCCAGCATCTC-3';

probe sequences: 5'-VIC-TGCCCCTCTATGACCTG-MGB-NFQ-3',

5'-FAM- CCCCTCTGTGACCTG-MGB-NFQ-3'.

*ESR1* Y537N: forward primer, 5'-CTGTACAGCATGAAGTGCAAGAAC-3',

reverse primer, 5'-TGGGCGTCCAGCATCTC-3';

probe sequences: 5'-VIC-TGGTGCCCCTCTATGAC-MGB-NFQ-3',

5'-FAM-TGCCCCTCAATGAC-MGB-NFQ-3'.

*ESR1* Y537S: forward primer, 5'-CAGCATGAAGTGCAAGAACGT-3',

reverse primer, 5'-TGGGCGTCCAGCATCTC-3';

probe sequences: 5'-VIC-CCCCTCTATGACCTGC-MGB-NFQ-3',

5'-FAM-CCCTCTCTGACCTGC-MGB-NFQ-3'.

*ESR1* D538G: forward primer, 5'-CAGCATGAAGTGCAAGAACGT-3',

reverse primer, 5'-TGGGCGTCCAGCATCTC-3';

probe sequences: 5'-VIC-CCCCTCTATGACCTGCT-MGB-NFQ-3',

5'-FAM-CCCTCTATGGCCTGCT-MGB-NFQ-3'.

*PIK3CA* E542K: forward primer, 5'-GGGAAAATGACAAAGAACAGCTCAA-3',

reverse primer, 5'-CTGTGACTCCATAGAAAATCTTTCTCCT-3';

probe sequences: 5'-VIC-CCTCTCTCTGAAATCA-MGB-NFQ-3',

5'-FAM-CCTCTCTCTAAAATCA-MGB-NFQ-3'.

*PIK3CA* E545K: forward primer, 5'-TCAAAGCAATTTCTACACGAGATCCT-3',

reverse primer, 5'-CTGTGACTCCATAGAAAATCTTTCTC-3';

probe sequences: 5'-VIC-CTCTCTGAAATCACTGAGCAG-MGB-NFQ-3',

5'- FAM-CTCTGAAATCACTAAGCAG-MGB-NFQ-3'.

*PIK3CA* H1047R: forward primer, 5'-GCAAGAGGCTTTGGAGTATTTCATG-3',

reverse primer, 5'-GCTGTTTAATTGTGTGGAAGATCCAA-3';

probe sequences: 5'-VIC-CCACCATGATGTGCATC-MGB-NFQ-3',

5'-FAM-CACCATGACGTGCATC-MGB-NFQ-3'.

#### *ESR1* mutations

Standard curves were prepared using *ESR1* mutant and wild-type plasmids ranging from 1000 copies/μL to 25 copies/μL. Mutation-specific TaqMan probes/primers (Life Technologies) were used in the PCR reaction, which had the following cycling conditions: 95 °C x 10 min (1 cycle), 40 cycles of 94 °C x 30 s and 56 °C x 1 min, and 10 °C hold.

#### *PIK3CA* mutations

Genomic DNA was extracted from the following cell lines: T84 (E542K heterozygous), MCF7 (E545K heterozygous), and HCT116 (H1047R heterozygous) using DNeasy Blood and Tissue Kit (Qiagen). Standard curves were prepared using genomic DNA from each of these cell lines with concentrations ranging from 1000 genome copies/μL to 10 genome copies/μL. Mutation-specific TaqMan probes/primers (Life Technologies) were used in the PCR reaction, which had the following cycling conditions: 95 °C x 10 min (1 cycle), 40 cycles of 94 °C x 30 s and 59 °C x 1 min, followed by 10 °C hold.

### Droplet digital PCR workflow

ddPCR was performed as previously described.^27^ Briefly, TaqMan PCR reaction mixtures were assembled from a 2 × ddPCR Mastermix (Bio-Rad) and custom 40x TaqMan probes/primers made specific for each assay. Twenty-five microliters of assembled ddPCR reaction mixture, which include either 5 μL of cfDNA sample or water as no template control was loaded into wells of a 96-well PCR plate. The heat-sealed PCR plate was subsequently loaded onto the Automated Droplet Generator (Bio-Rad). After droplet generation, the new 96-well PCR plate was heat-sealed, placed on a conventional thermal cycler, and amplified to the end-point. After PCR, the 96-well PCR plate was read on the QX100 droplet reader (Bio-Rad). Analysis of the ddPCR data was performed with QuantaSoft analysis software (Bio-Rad) that accompanied the droplet reader.

### Cell lines

MCF7 cells were purchased from ATCC. The cells were authenticated and regularly tested for mycoplasma contamination. The cells were maintained in DMEM supplemented with 10% heat-inactivated fetal bovine serum (FBS) and 1% penicillin/streptomycin (P/S). For hormone-depleted (HD) conditions, cells were kept in phenol-red free medium supplemented with 10% heat-inactivated charcoal-stripped (CS)-FBS and 1% P/S. All cells were incubated at 37 °C in 5% CO_2_.

For the cell proliferation assays the cells were plated in 24-well plates (2.5 × 10^4^/well). The cells were trypsinized and collected at the noted time points. The number of viable cells was determined by Trypan blue exclusion staining and manually counted with a hemocytometer using independent triplicates.

### Generation of the *ESR1* mutant knocked-in cells

The generation of the *ESR1* Y537S and D538G knock-in mutant cells in MCF7 cells was described previously.^14^ The *ESR1* E380Q knock-in cells in MCF7 cells were generated using a similar protocol. The TALENs were designed to target intron 4 and the TALEN recognition sequences were:

XTN1: (bold = TAL binding sites, non-bold = cut region)

5' **TGCTCCTAACTTGCTCT**tggacaggtaagtgacct**GGCTGTAGCTTAGGAGTA** 3'

XTN2: (bold = TAL binding sites, non-bold = cut region)

5' **TAACTTGCTCTTGGACA**Ggtaagtgacctggct**GTAGCTTAGGAGTAGCA** 3'

The sequences were synthesized and cloned into the SQT281 vector (Transposagen) that includes a Fok1 nuclease. For the homologous recombination we used a donor vector that contained the targeting constructs of *piggyBac* transposon and the a puromycin-thymidine kinase selection cassette flanked by about 500 bp of ESR1 genomic sequence with the coding changes (GAA > CAA) in the 3' end matching the exon 5 coding region. The MCF7 cells were transiently transfected with the TALEN vector and donor vector using Lipofectamine 2000 (Invitrogen). After puromycin selection, clones with the desired mutation were detected by Sanger sequencing and transiently transfected with transposase expression plasmids (Transposagen) for removal of the selection cassettes followed by gancyclovir treatment for negative selection. We confirmed the mutations by Sanger sequencing and RNA-sequencing.

### RNA-seq

Total RNA was isolated using an RNeasy Mini Kit (Qiagen). For all cells and conditions samples were done in triplicates. RNA-seq libraries were made using the TruSeq RNA Sample Preparation Kit (Illumina) adapted for use on the Sciclone (Perkin-Elmer) liquid handler. Samples were sequenced on an Illumina Nextseq500. Alignment to the hg19 human genome was done using STAR v2.5.1^28^ followed by Transcript assembly using cufflinks v2.2.1^29^ and quality control steps were done using STAR v2.5.1 and RseQC v2.6.2^30^.

### Statistical analysis

Descriptive statistics were used to describe the disease and treatment characteristics of patients with and without an *ESR1* mutation or a *PIK3CA* mutation. The association between the emergence of the *ESR1* mutations in cfDNA, clinicopathological features and treatments at the time of the early-stage and metastatic disease was assessed using Fisher’s exact test. A Wilcoxon rank sum test was conducted to test association between time of disease development and mutation status. The sensitivity and specificity of mutation detection in plasma and metastatic tumor samples were calculated with 95% confidence interval. Analyses were performed using R version 3.3.1. (www.r-project.org). The Student’s *t*-test was used for the analysis of the cell line proliferation studies. The IC50 was calculated using GraphPad PRISM.

### Data availability

All RNA-seq data was uploaded to GEO, accession number GSE112243. All other data are available from the corresponding author upon reasonable request.

## Electronic supplementary material

Figure S1
