# Supplementary figures and images for: Unraveling the clinicopathological features driving the emergence of ESR1 mutations in metastatic breast cancer
Source: NPJ Breast Cancer. 2018 Aug 2;4:22. doi: 10.1038/s41523-018-0075-5 (PMC6072793; doi:10.1038/s41523-018-0075-5)

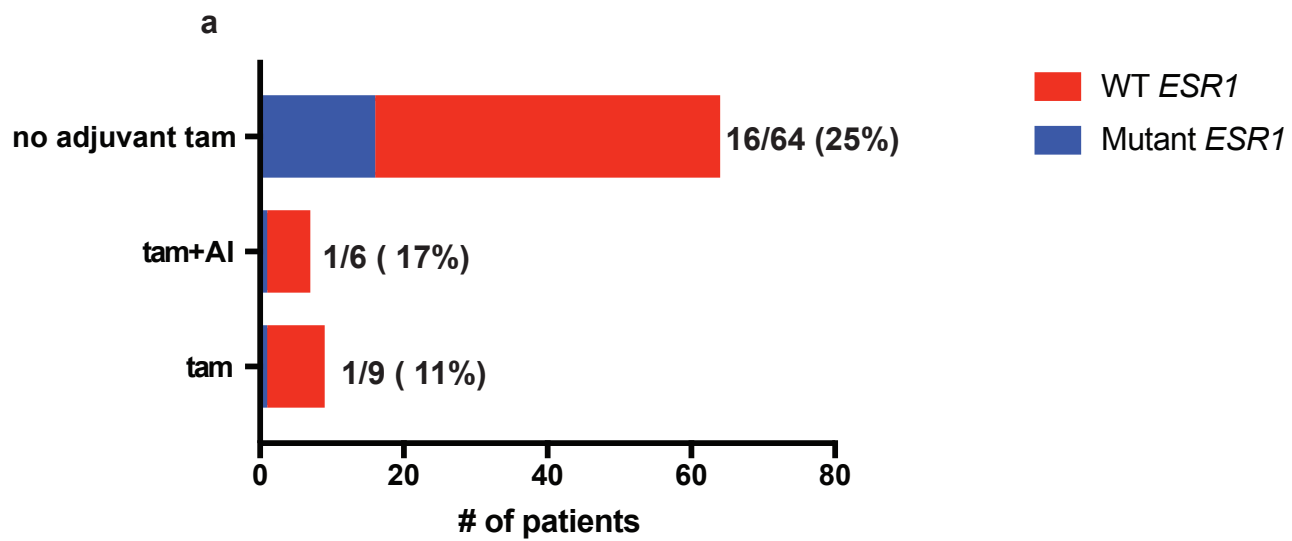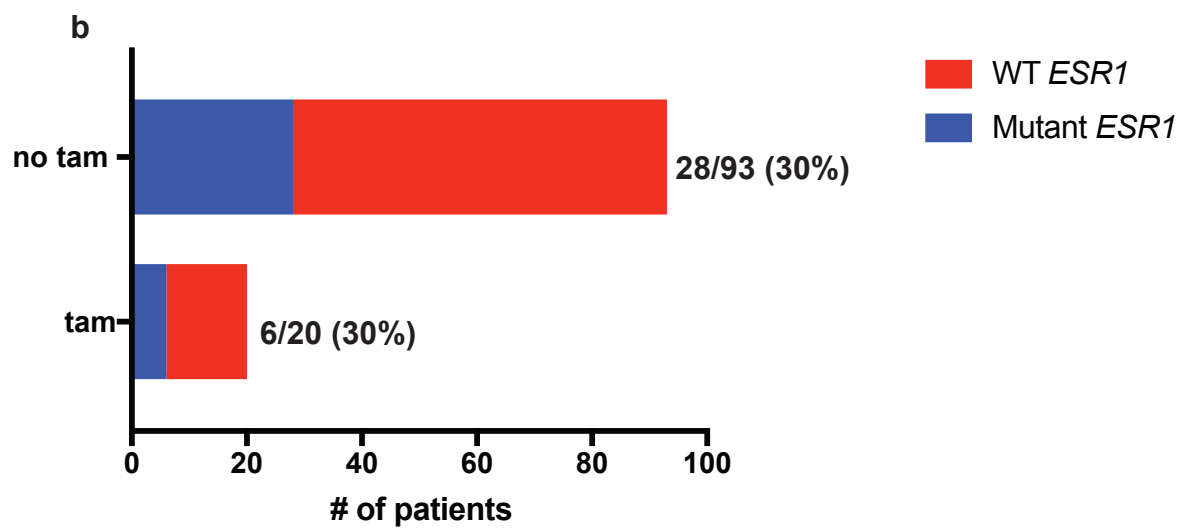

Supplement: Supplementary file 1 — Figure S1 [file 41523_2018_75_MOESM1_ESM.pdf]
